# Loss of periostin ameliorates adipose tissue inflammation and fibrosis *in vivo*

**DOI:** 10.1038/s41598-018-27009-9

**Published:** 2018-06-04

**Authors:** Fumiko Nakazeki, Masataka Nishiga, Takahiro Horie, Hitoo Nishi, Yasuhiro Nakashima, Osamu Baba, Yasuhide Kuwabara, Tomohiro Nishino, Tetsushi Nakao, Yuya Ide, Satoshi Koyama, Masahiro Kimura, Shuhei Tsuji, Naoya Sowa, Shigeo Yoshida, Simon J. Conway, Motoko Yanagita, Takeshi Kimura, Koh Ono

**Affiliations:** 1 Department of Cardiovascular Medicine, Graduate School of Medicine, Kyoto University, Kyoto, 606-8507 Japan; 2 Department of Ophthalmology, Kyushu University Graduate School of Medical Sciences, Fukuoka, 812-8582 Japan; 3 Herman B Wells Center for Pediatric Research, Indiana University of Medicine, Indianapolis, Indiana, USA; 4 Department of Nephrology, Graduate School of Medicine, Kyoto University, Kyoto, 606-8507 Japan

## Abstract

Recent evidence suggests that the accumulation of macrophages as a result of obesity-induced adipose tissue hypoxia is crucial for the regulation of tissue fibrosis, but the molecular mechanisms underlying adipose tissue fibrosis are still unknown. In this study, we revealed that periostin (*Postn*) is produced at extraordinary levels by adipose tissue after feeding with a high-fat diet (HFD). *Postn* was secreted at least from macrophages in visceral adipose tissue during the development of obesity, possibly due to hypoxia. *Postn*^−/−^ mice had lower levels of crown-like structure formation and fibrosis in adipose tissue and were protected from liver steatosis. These mice also showed amelioration in systemic insulin resistance compared with HFD-fed WT littermates. Mice deficient in *Postn* in their hematopoietic compartment also had lower levels of inflammation in adipose tissue, in parallel with a reduction in ectopic lipid accumulation compared with the controls. Our data indicated that the regulation of *Postn* in visceral fat could be beneficial for the maintenance of healthy adipose tissue in obesity.

## Introduction

A gradual and healthy expansion of adipose tissue in response to caloric intake is associated with appropriate vascular and extracellular matrix (ECM) remodeling^1^. In contrast, increased adiposity is likely to involve a state of chronic inflammation in adipose tissue, thereby inducing systemic insulin resistance and ectopic lipid accumulation.

Previous studies showed that macrophages are crucial in shifting adipose tissue inflammation and ECM remodeling towards a pathological state versus an adaptive response. Macrophages in lean adipose tissue secrete anti-inflammatory cytokines, which contribute to the maintenance of tissue homeostasis^2–5^. However, M1-like macrophages, which produce pro-inflammatory mediators such as tumor necrosis factor α (TNF-α), accumulate in adipose tissue and promote insulin resistance in conditions of obesity^2,3^. It is conceivable that increased adipose tissue inflammation stimulates adipocyte lipolysis and tissue fibrosis, and thus enhances the release of free fatty acids, which may accumulate in non-adipose tissues as ectopic fat^6,7^. Although recent evidence suggests that the accumulation of macrophages resulting from obesity-induced adipose tissue hypoxia are crucial for the regulation of tissue fibrosis^8–10^, the molecular mechanisms underlying adipose tissue fibrosis are still unknown.

Periostin (*Postn*) is a secreted ECM/matricellular protein that contains 4 fasciclin domains homologous to the insect protein fasciclin I, which is involved in cell adhesion^11,12^. At baseline, low levels of *Postn* were detected in various adult tissues. On the other hand, *Postn* was induced by various cytokines and can sustain or amplify inflammation. It is also known that *Postn* is secreted as a latent consequence of inflammatory responses rather than regulating its processes^13^. In addition, recent work has shown that hypoxia-inducible *Postn* can act as a chemoattractant for tumor-associated macrophage recruitment in a mouse model of glioblastoma^14,15^. Another role of *Postn* has been defined in many tissues, where the loss of *Postn* results in decreased collagen cross-linking and degradation of the ECM^16–18^. *Postn* has been shown to be linked to several inflammatory diseases, including atherosclerosis, asthma, and skin inflammation as well as malignant diseases such as breast, colon, head and neck, pancreatic, lung, gastric, and hepatocellular carcinoma^19^. In the case of metabolic disease, there is experimental evidence implicating *Postn* in hepatic steatosis, inflammation, and fibrosis^20^. It was also reported that hepatic *Postn* expression increased eight fold in mice fed a high-fat diet (HFD) compared with a standard diet (SD)^20^.

Here, we revealed that *Postn* is produced in extraordinary levels from adipose tissue after HFD feeding, and the protein level of *Postn* was ~80 times higher than that observed in the liver. The source of *Postn* was at least from macrophages in visceral adipose tissue during the development of obesity. Hypoxia increased *Postn* expression in macrophages, resulting in further recruitment of macrophages. *Postn*-deficient (*Postn*^−/−^) mice were resistant to obesity-induced crown-like structure (CLS) formation and adipose tissue fibrosis. Less fibrosis was accompanied with the expansion of adipocyte size and reduced ectopic lipid accumulation compared to wild-type (WT) mice. Thus, *Postn*^−/−^ mice showed amelioration in systemic insulin resistance compared with HFD-fed WT littermates (*Postn*^+/+^). Mice deficient in *Postn* in their hematopoietic compartment were also resistant to ectopic lipid accumulation, in parallel with a marked reduction in inflammation in adipose tissue.

## Results

### Periostin expression in adipose tissues during obesity

**Figure 1:**
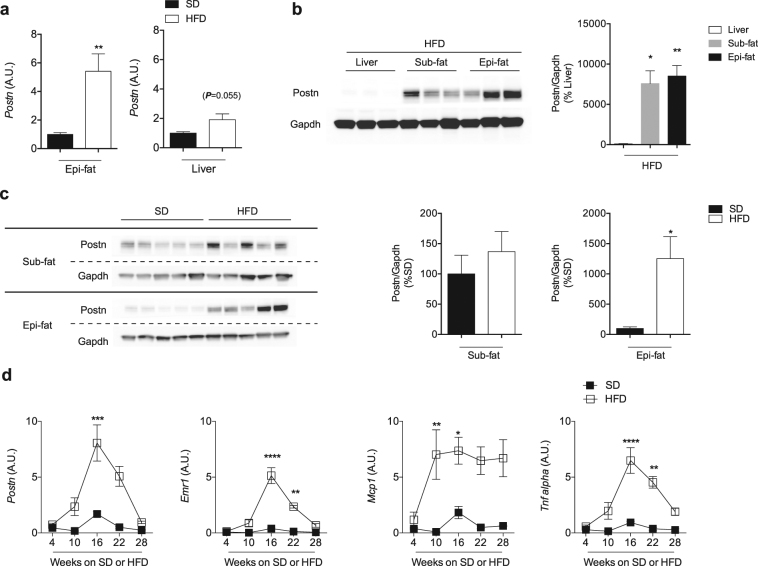
Periostin (*Postn*) is dramatically upregulated in adipose tissue during obesity. (**a**) mRNA expression levels of *Postn* in the liver and adipose tissue (epididymal fat [Epi-fat]) of C57BL/6J male mice after SD or HFD feeding for 16 weeks. n = 5 in each group. **p < 0.01 by unpaired t-test. (**b**) Protein levels of Postn in the liver and adipose tissue (subcutaneous fat [Sub-fat]) from diet-induced obese mice after 16 weeks of HFD feeding. n = 5, *p < 0.05, **p < 0.01 by one-way ANOVA. (**c**) Protein levels of Postn in adipose tissue from lean and obese mice after 16 weeks of SD or HFD feeding, respectively. n = 5, *p < 0.05 by unpaired t-test. (**d**) Time course of *Postn* and pro-inflammatory gene profiling in Epi-fat of WT SD-fed or HFD-fed mice for up to 36 weeks. n = 3 to 6, *p < 0.05, **p < 0.01, ***p < 0.001, ****p < 0.0001 versus SD at each time point. Statistical comparisons were made by unpaired t-test.

To explore the role of *Postn* during obesity, we analyzed *Postn* expression in the liver and adipose tissue (subcutaneous fat [Sub-fat] and epididymal fat [Epi-fat]) from C57BL/6 J male mice. Eight-week-old mice were fed either SD or HFD for up to 30 weeks. Body and liver weights of mice gradually increased over the duration of HFD feeding, but adipose tissue weights peaked after 10–16 weeks of HFD feeding (Supplementary Fig. 1a). Histological examination revealed that liver steatosis and Epi-fat inflammation were evident after HFD feeding for 16 weeks. However, there was little change in Sub-fat tissues (Supplementary Fig. 1b). After 16 weeks of HFD feeding, *Postn* mRNA expression was higher in Epi-fat tissue than in the liver (Fig. 1a). Postn protein levels in Epi-fat were approximately 80 times higher than those observed in the liver (Fig. 1b). A marked induction of Postn in Epi-fat compared with Sub-fat by HFD feeding suggested a specific role of *Postn* in visceral fat during obesity (Fig. 1c).

Next, we examined the time course of *Postn* expression in Epi-fat, and it was found that *Postn* mRNA levels changed roughly in parallel with inflammatory gene expression, including expression of *Emr1*, *Mcp1*, and *Tnf-α* (Fig. 1d). Of note, interstitial fibrosis in Epi-fat decreased gradually after 16 weeks of HFD feeding (Supplementary Fig. 1c) in parallel with down-regulation of *Postn* and inflammatory gene expression levels (Fig. 1d).

### Altered lipid distribution in *Postn*^−/−^ mice

**Figure 2:**
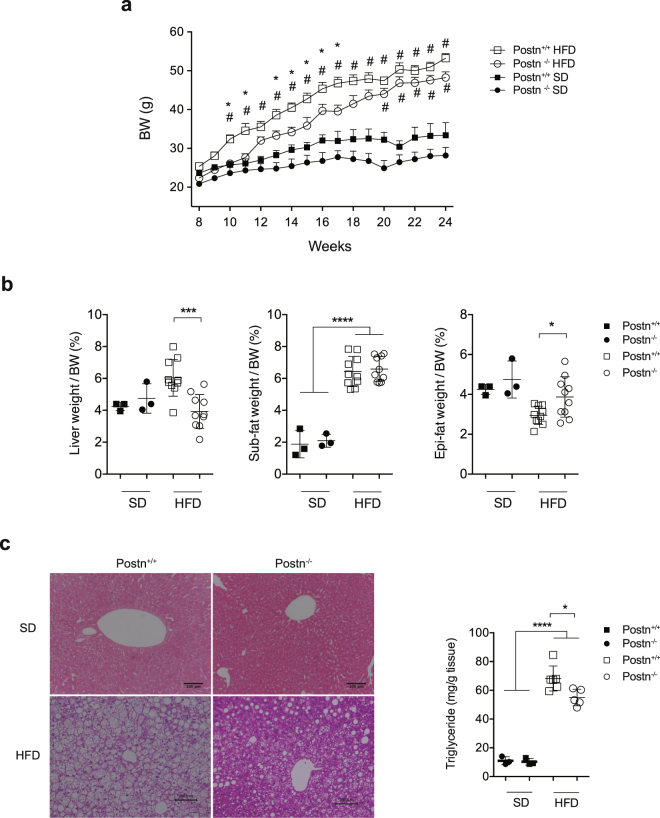
Altered lipid distribution in *Postn*^−/−^ mice. (**a**) Development of body weight of *Postn*^+/+^ and *Postn*^−/−^ male SD-fed or HFD-fed mice. n = 5 in the SD groups, n = 11–13 in the HFD groups, *p < 0.05 versus HFD-fed *Postn*^+/+^ mice, ^#^p < 0.05 versus SD-fed *Postn*^+/+^ mice by one-way ANOVA. (**b**) Liver and adipose tissue weights normalized to whole body weight of *Postn*^+/+^ and *Postn*^−/−^ SD-fed or HFD-fed mice. n = 3 for the SD groups, n = 10 for the HFD groups. *p < 0.05, **p < 0.01, ***p < 0.001, ****p < 0.0001 by one-way ANOVA. (**c**) Left, representative microscope images of the livers of *Postn*^+/+^ and *Postn*^−/−^ SD-fed or HFD-fed mice. Scar bars, 100 μm. Right, triglyceride levels in the livers of *Postn*^+/+^ and *Postn*^−/−^ mice. n = 3 for the SD groups, n = 5 for the HFD groups. *p < 0.05, ****p < 0.0001 by one-way ANOVA.

**Figure 3:**
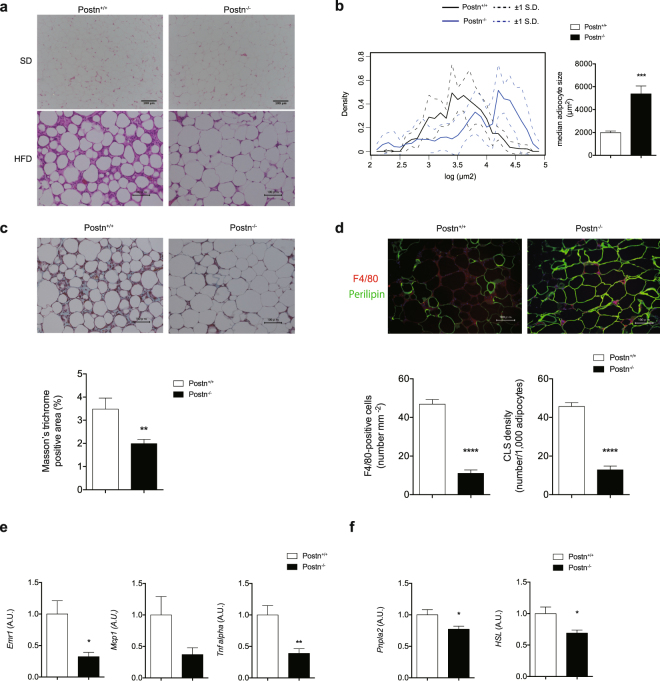
Ameliorated inflammation of adipose tissue in *Postn*^−/−^ mice. (**a**) Representative H&E staining images of adipose tissue (Epi-fat) of *Postn*^+/+^ and *Postn*^−/−^ SD-fed or HFD-fed mice. (**b**) Left, density curve of adipocyte surface areas in Epi-fat. Right, median adipocyte cell size in Epi-fat of *Postn*^+/+^ and *Postn*^−/−^ HFD-fed mice. ***p < 0.001 by unpaired t-test. n = 3 images per mouse, 10 mice each. (**c**) Top, representative Masson’s trichrome staining images of adipose tissue (Epi-fat) of *Postn*^+/+^ and *Postn*^−/−^ HFD-fed mice. Bottom, quantification of Masson’s trichrome-positive area (identical with interstitial fibrosis). **p < 0.01 by unpaired t-test. (**d**) Top, representative immunofluorescent staining of F4/80 (red) and perilipin (green). Bottom, the number of F4/80-positive cells and crown-like structure (CLS) density in Epi-fat of *Postn*^+/+^ and *Postn*^−/−^ mice after HFD feeding for 16 weeks. Scale bars, 50 μm. ****p < 0.0001 by unpaired t-test. (**e**) Pro-inflammatory (*Emr1, Mcp1* and *Tnf-α*) mRNA levels in Epi-fat from HFD-fed *Postn*^+/+^ and *Postn*^−/−^ mice. n = 6 each, *p < 0.05, **p < 0.01 by unpaired t-test. (**f**) Lipolysis associated mRNA levels (*Pnpla2* and *Hsl*) mRNA levels in Epi-fat from HFD-fed *Postn*^+/+^ and *Postn*^−/−^ mice. n = 6 each, *p < 0.05 by unpaired t-test.

Eight-week-old male C57BL/6J mice that had undergone systemic genetic ablation of *Postn* and age- and sex-matched WT littermates were fed either SD or HFD for 16 weeks. The weight of SD-fed *Postn*^−/−^ mice tended to be reduced throughout the experimental period. Although the body weight of HFD-fed *Postn*^−/−^ mice was lower in the early phase (Fig. 2a), it caught up with that of WT littermates after 10 weeks of HFD feeding (18 weeks of age). There was no difference in HFD intake for a week (Supplementary Fig. 2a). After 16 weeks of HFD feeding, the Epi-fat weight normalized to body weight was increased with a reciprocal reduction in liver weight in *Postn*^−/−^ mice relative to WT littermates after HFD feeding (Fig. 2b). Epi-fat *Postn* levels were increased both at the mRNA and protein levels (Supplementary Fig. 2b and c). Histological examination revealed that hepatic steatosis and interstitial fibrosis in Epi-fat were markedly attenuated in *Postn*^−/−^ mice compared with WT littermates (Figs 2c, 3a–c). Consistent with this, hepatic triglyceride content and serum aspartate aminotransferase (AST) and alanine aminotransferase (ALT) levels were significantly reduced in *Postn*^−/−^ mice compared with WT littermates (Fig. 2c, Supplementary Fig. 2c). On the other hand, there were no apparent differences in T-cho, LDL-C, HDL-C, TG or NEFA after 16 weeks of HFD feeding between these two genotypes of mice (Supplementary Fig. 2d). However, when we observed serial changes in organ weight after HFD feeding, Epi-fat weight peaked at 10 weeks after HFD feeding and liver weight is increased gradually until 22 weeks on a HFD (Supplementary Fig. 1a). Similar changes were also observed in the histology of the liver and adipose tissue (Supplementary Fig. 1c). These results also suggested that changes in adipose tissue can affect lipid distribution in the liver. It is known that *Postn*^−/−^ mice showed specifically impaired pancreatic regeneration in islet β-cells^21^. However, pancreatic regeneration was not observed after HFD feeding and it was not related with our findings in *Postn*^−/−^ mice. Actually, the islet size in *Postn*^−/−^ mice was relatively smaller than that of WT mice (Supplementary Fig. 2e), which may indicate the amelioration of insulin resistance in *Postn*^−/−^ mice compared with WT mice. All of these results demonstrated that the loss of Postn affected lipid distribution between liver and Epi-fat tissues. Concerning to the Postn receptors, we measured the expression levels of *Itgav*, *Itga6*, and *Itgb3* in Epi-fat and liver. As shown in Supplementary Fig. 2f, Itga*v* and *Itgb3* were reduced in *Postn*^−/−^ mice Epi-fat, which may indicate the reduced Postn signaling.

### Reduced CLS formation and adipose tissue fibrosis in *Postn*^−/−^ mice

Histological examination revealed that adipocyte cell size from Epi-fat and Sub-fat in HFD-fed mice was larger in *Postn*^−/−^ mice than in WT littermates (Fig. 3a,b, Supplementary Fig. 3a,b). In addition, Masson’s trichrome staining showed extensive interstitial fibrosis in Epi-fat of WT littermates, which was markedly suppressed in *Postn*^−/−^ mice (Fig. 3c).

We next examined the number of CLSs in Epi-fat by double-immunofluorescence staining with anti-F4/80 (red) and anti-Perilipin (green) antibodies. The number of F4/80-positive cells and the density of CLSs in the Epi-fat of HFD-fed *Postn*^−/−^ mice were markedly reduced compared with those of HFD-fed WT littermates (Fig. 3d). Consistent with these staining results, flow cytometric analysis revealed that the number of F4/80^+^ cells isolated from Epi-fat was lower in HFD-fed *Postn*^−/−^ mice than in HFD-fed WT littermates (Supplementary Fig. 3c,d). Moreover, there was a significant decrease in pro-inflammatory gene expression levels, such as *Emr1*, *Mcp1*, and *Tnf-α* in Epi-fat of *Postn*^−/−^ mice than in those of WT littermates (Fig. 3e). In addition, *Pnpla2* and *Hsl* expressions were significantly reduced in *Postn*^−/−^ mice compared with WT littermates, which indicated that reduced lipolysis may have attenuated the altered lipid distribution in the liver of *Postn*^−/−^ mice (Fig. 3f).

### *Postn*^−/−^ mice show ameliorated insulin resistance *in vivo*

**Figure 4:**
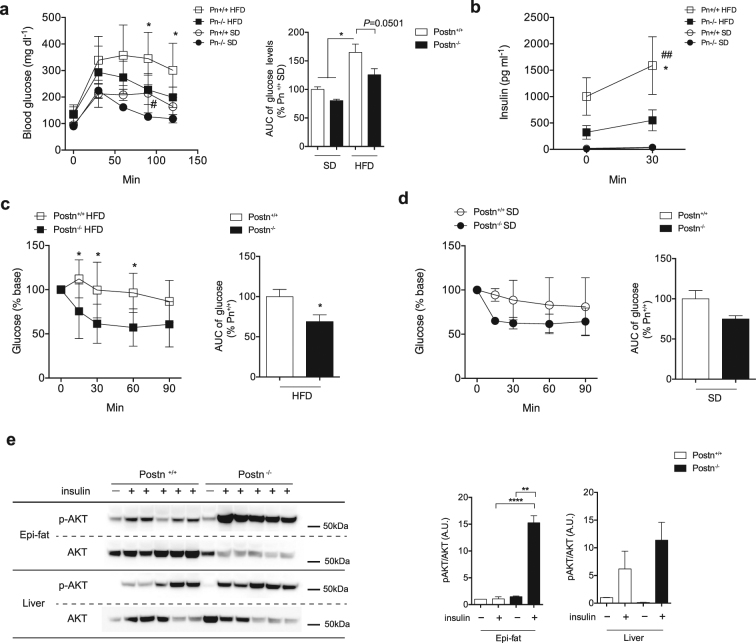
Ameliorated glucose metabolism in *Postn*^−/−^ mice. (**a**) Serial changes in glucose levels and area under the curve (AUC) of glucose levels after intraperitoneal injection of glucose in *Postn*^+/+^ and *Postn*^−/−^ SD-fed or HFD-fed mice at 12 weeks for HFD. n = 3 for the SD groups, n = 8–9 for the HFD groups. *p < 0.05 versus *Postn*^−/−^ mice fed HFD by one-way ANOVA. (**b**) Serial changes in insulin levels after intraperitoneal injection of glucose in *Postn*^+/+^ and *Postn*^−/−^ SD-fed or HFD-fed mice at 14 weeks for HFD. n = 4 for the SD groups, n = 6 for the HFD groups. ^##^p < 0.01 versus *Postn*^+/+^ SD-fed mice. *p < 0.05 versus Postn^−/−^ HFD-fed mice by one-way ANOVA. (**c**) Serial changes in glucose levels and AUC of glucose levels after intraperitoneal injection of insulin in *Postn*^+/+^ and *Postn*^−/−^ HFD-fed mice at 16 weeks for HFD. n = 5–6 each, analyzed by unpaired t-test. (**d**) Serial changes in glucose levels and AUC of glucose levels after intraperitoneal injection of insulin in *Postn*^+/+^ and *Postn*^−/−^ SD-fed mice. n = 4 each, analyzed by unpaired t-test. (**e**) Protein levels of phosphorylated Akt (Thr308) in Epi-fat and liver. *Postn*^+/+^ and *Postn*^−/−^ HFD-fed mice were injected with saline or insulin via the postcaval vein. n = 3 for saline, n = 5 for insulin. *p < 0.05, ****p < 0.0001 versus *Postn*^+/+^ insulin by unpaired t-test.

We next examined the effect of *Postn* deficiency on glucose homeostasis in HFD-fed mice. We performed intraperitoneal (IP) glucose tolerance tests (GTTs) after 12 weeks of SD or HFD feeding and found that *Postn*^−/−^ mice showed better glucose tolerance than HFD-fed WT littermates (Fig. 4a). The insulin response during the GTTs was significantly lower in HFD-fed *Postn*^−/−^ mice versus WT littermates (Fig. 4b). ITTs after 14 weeks of HFD feeding showed that HFD-fed *Postn*^−/−^ mice were more insulin sensitive than WT littermates (Fig. 4c). There was no significant difference between these mice when they were fed SD (Fig. 4d). Moreover, the insulin-induced phosphorylation of AKT was significantly increased in the Epi-fat of *Postn*^−/−^ mice compared with that of WT littermates, and the same tendency was observed in the liver in *Postn*^−/−^ mice compared with that of WT littermates (Fig. 4e).

### *Postn* is induced in Epi-fat tissue macrophages by obesity

**Figure 5:**
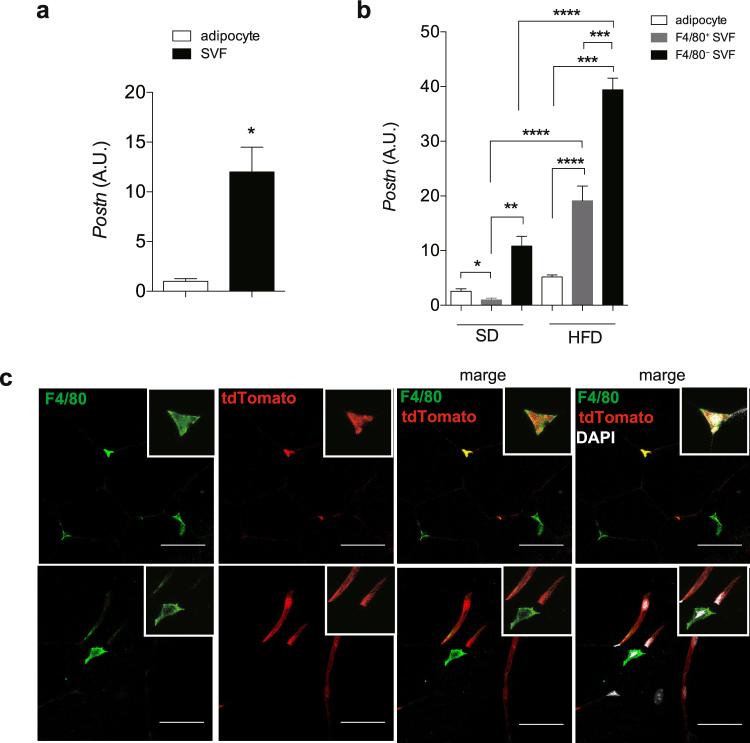
Postn is secreted by the stromal vascular fraction (SVF). (**a**) mRNA expression of *Postn* in adipose tissue (Epi-fat) from C57BL/6J male HFD-fed mice. n = 3, *p < 0.05 by unpaired t-test. (**b**) mRNA expression of *Postn* in adipocytes, F4/80^−^ and F4/80^+^ cells, isolated from SVF. n = 3 each, *p < 0.05, **p < 0.01, ***p < 0.001, ****p < 0.0001 by one-way ANOVA. (**c**) Representative immunofluorescent staining of F4/80 (green) in Epi-fat from obese *tdTomato* fluorescent protein reporter mice demonstrated *Postn* expressing cells. Scar bars, 50 μm.

To identify the cellular sources of *Postn*, we isolated adipocytes and the stromal vascular fraction (SVF), which was further divided into F4/80^−^ cells and F4/80^+^ cells, from the Epi-fat of lean and obese mice (Supplementary Fig. 4a). *Postn* mRNA levels were significantly higher in SVF than that in adipocytes (Fig. 5a). When cells were divided into subfractions, *Postn* mRNA levels were significantly higher in F4/80^−^ cells than in other fractions both with SD and HFD feeding. Although *Postn* mRNA levels were lower in F4/80^+^ cells than in any of the other cell fractions from the Epi-fat of SD-fed mice, it was remarkably increased in HFD-fed mice (Fig. 5b), suggesting a requirement for HFD feeding for the activation of Postn production in macrophages. In addition, myofibroblast marker (*αSMA*) and preadipocyte marker (*Pdgfr-α* and *Ppar-γ*) were activated in F4/80^−^ cells of HFD-fed mice (Supplementary Fig. 4b).

To confirm which cells produce *Postn*, we utilized *Rosa26-loxP-stop-loxP-tdTomato* transgenic mice crossed with *Periostin-Cre* mice. *tdTomato* is activated by *Postn*-specific promoter-regulated *cre* expression (Supplementary Fig. 4c). After HFD feeding for 24 weeks, when obesity-induced histological changes such as enlargement of adipocyte sizes were prominent, *tdTomato*-positive cells were both positive and negative for anti-F4/80 antibody immunofluorescent staining (Fig. 5c). tdTomato expression did not overlap with perilipin staining, which indicates that a portion of Postn-positive cells are interstitial macrophages (Supplementary Fig. 4d).

### Loss of *Postn* reduces macrophage migration *in vitro*

**Figure 6:**
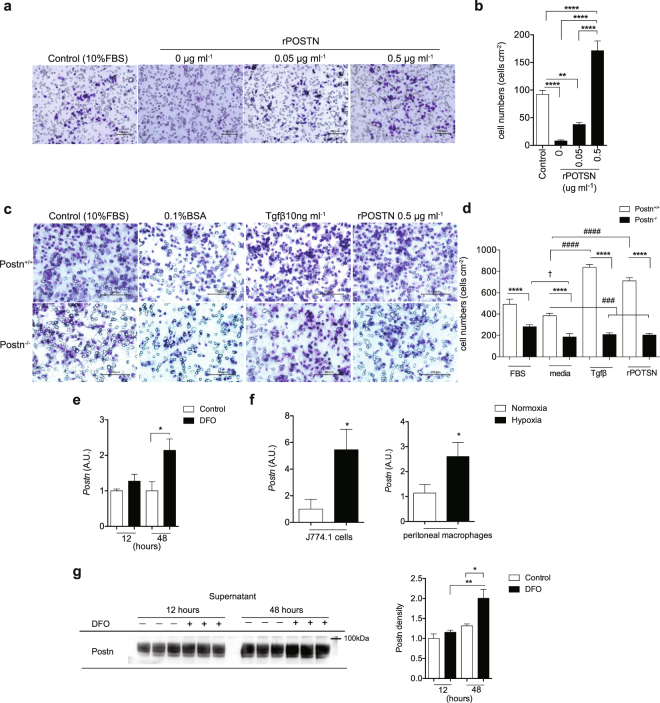
Postn regulates macrophage migration. (**a**) Representative images of J774.1 cells migrating toward rPOSTN in Transwell assays. Scale bar, 100 μm. (**b**) Graphical analysis of e showing the migration of J774.1. n = 10–15 fields, **p < 0.01, ****p < 0.0001 by one-way ANOVA. (**c**) Representative images of *Postn*^+/+^ and *Postn*^-/-^ peritoneal macrophages migrating toward transforming growth factor-β (TGF-β) or rPOSTN in trans-well assays. Scale bar, 100 μm. (**d**) Graphical analysis of g showing the migration of peritoneal macrophages. n = 8–12 fields for *Postn*^+/+^, n = 21–22 fields for *Postn*^−/−^, ****p < 0.001 versus *Postn*^+/+^ at each condition, ^###^p < 0.01, ^####^p < 0.0001 versus Postn^+/+^ media, ^†^p < 0.05 versus *Postn*^−/−^ media by one-way ANOVA. (**e**) mRNA expression of *Postn* in J774.1 cells with or without deferoxamine mesylate (DFO) treatment over the time course. n = 6, *p < 0.05 by one-way ANOVA. (**f**) mRNA expression of *Postn* in J774.1 cells and peritoneal macrophages that were exposed to hypoxia. n = 5 for J774.1, n = 5–7 for peritoneal macrophages, *p < 0.05 by unpaired t-test. (**g**) Protein levels of Postn in J774.1 cells supernatants with or without DFO treatments over the time-course. n = 3 each, *p < 0.05, **p < 0.01 by one-way ANOVA.

A previous study showed that *Postn* can act as a chemoattractant for macrophages in a model of glioblastoma^14^. Actually, the migration of J774.1 macrophages towards recombinant POSTN (rPOSTN) was significantly enhanced in accordance with the increase in rPOSTN protein concentration (Fig. 6a,b). Moreover, the migration ability of *Postn*^−/−^ macrophages was significantly impaired compared with that of WT macrophages. In addition, both TGF-β1 and rPOSTN caused a significant increase in the migration of WT macrophages but not in *Postn*^−/−^ macrophages. (Fig. 6c,d). These observations indicated a fundamental defect in the ability of macrophages to migrate in *Postn*^−/−^ mice.

### Hypoxia promotes *Postn* secretion in macrophages

Recent reports suggested that hypoxia is a new potential risk factor for chronic inflammation in obesity^22,23^. Infiltrated macrophages were predominantly found in hypoxic areas in the adipose tissue of obese mice^24^. To determine whether hypoxia induced an increase in the expression of *Postn* in macrophages, we first treated the J774.1 macrophage cell line with deferoxamine mesylate, which mimics hypoxia, for 12 or 48 hours. We found that *Postn* expression was elevated in hypoxia mimetic conditions in parallel with genes downstream of hypoxia-inducible factor (HIF), such as *Glut1* and *Pgk1* (Fig. 6e, Supplementary Fig. 5a). Similar to this observation, *Postn* expression was enhanced in both J774.1 cells and peritoneal macrophages exposed to hypoxic conditions (5% O2) (Fig. 6f, Supplementary Fig. 5b).

Because Postn is a cell-secreted protein, we incubated J774.1 cells in hypoxia mimetic conditions for 12 or 48 hours to obtain hypoxia-stimulated J774.1 cell supernatants. We found that Postn protein levels in the supernatants increased in hypoxia mimetic conditions (Fig. 6g,h). In contrast, protein levels of Postn inside the cells did not change (Supplementary Fig. 5c).

### *Postn* deletion in hematopoietic cells reduced obesity-induced adipose tissue inflammation

Considering the role of macrophages in the propagation of inflammatory signals in adipose tissue, we hypothesized that deletion of *Postn* in hematopoietic-derived cells, which include macrophages, could reduce obesity-related increases in macrophage infiltration and inflammation and subsequently prevent *in vivo* ectopic lipid accumulation and insulin resistance. To address this hypothesis, we generated mice with *Postn* deleted exclusively in hematopoietic cells. Mice transplanted with bone marrow (BM) from *Postn*^−/−^ mice (BM-*Postn*^−/−^) were deficient in *Postn* in hematopoietic-derived cells. Mice transplanted with bone marrow from WT littermates (BM-*Postn*^+/+^) displayed normal *Postn* expression in all hematopoietic and nonhematopoietic cells/tissues (Supplementary Fig. 6a).

**Figure 7:**
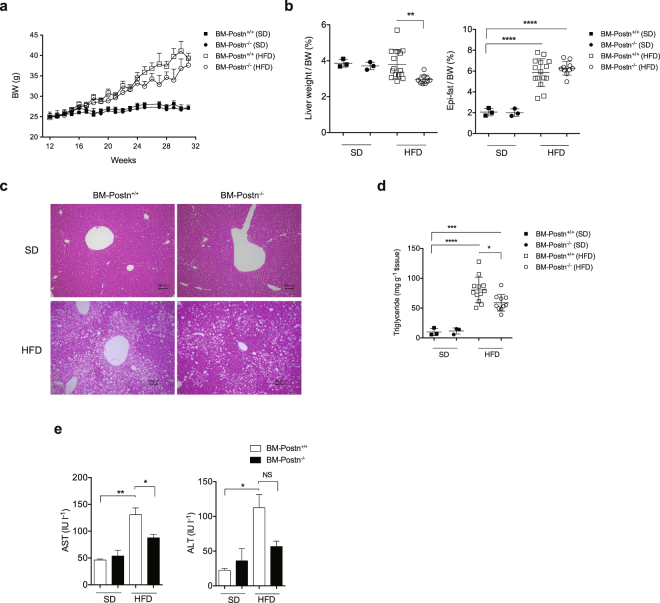
Improved liver steatosis in BM-*Postn*^−/−^ mice. (**a**) Development of body weight of SD-fed or HFD-fed mice over 30 weeks after BM transplantation. n = 3 for the SD groups, n = 11–15 for the HFD groups, analyzed by one-way ANOVA at each time point. (**b**) Liver and adipose tissue weights of BM transplantation recipient *Postn*^+/+^ SD-fed or HFD-fed mice transplanted with *Postn* WT hematopoietic cells (BM-*Postn*^+/+^) and *Postn* deletion mutant hematopoietic cells (BM-*Postn*^−/−^). n = 3 for the SD groups, n = 11–15 for the HFD groups. *p < 0.05, **p < 0.01 by one-way ANOVA. (**c**) Representative microscope images of the livers of BM transplanted SD-fed or HFD-fed mice. Scar bars, 100 μm. (**d**) Triglyceride levels in the livers of BM transplanted mice. n = 3 for the SD groups, n = 9 for the HFD groups. *p < 0.05, ***p < 0.001, ****p < 0.0001 by one-way ANOVA. (**e**) Serum profile of BM transplanted SD-fed or HFD-fed mice. n = 3 for the SD groups, n = 11–15 for the HFD groups, *p < 0.05, **p < 0.01, ****p < 0.001 analyzed by unpaired t-test.

As expected, body weight gain in HFD-fed mice significantly outpaced SD-fed mice. There were no significant differences in body weight between BM-*Postn*^+/+^ and BM-*Postn*^−/−^ mice (Fig. 7a). We did not observe differences in absolute body weight or adipose tissue weight between these two groups. However, HFD-fed BM-*Postn*^−/−^ mice had lower liver weight per body weight compared with BM-*Postn*^+/+^ mice (Fig. 7b). *Postn* mRNA and protein levels were lower in Epi-fat from HFD-fed BM-*Postn*^−/−^ mice than in those from BM-*Postn*^+/+^ mice, although *Postn* mRNA levels in the liver were not different between the two groups (Supplementary Fig. 6b,c).

Histological examination revealed that hepatic steatosis was markedly attenuated in BM*-Postn*^−/−^ mice relative to WT littermates (Fig. 7c). Consistent with this, hepatic triglyceride content and serum AST and ALT levels were significantly reduced in BM*-Postn*^−/−^ mice compared with those in BM-*Postn*^+/+^ (Fig. 7d,e).

**Figure 8:**
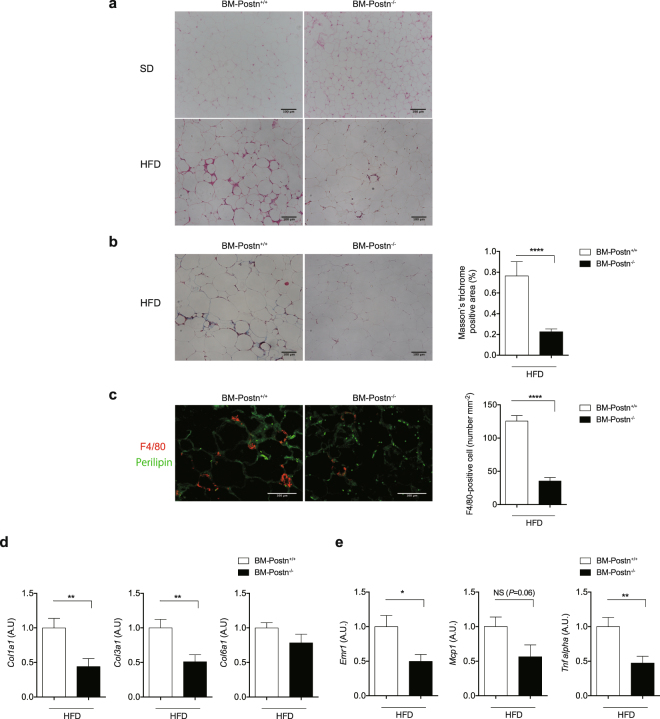
Hematopoietic cell-specific *Postn* deletion altered adipose tissue remodeling. (**a**) Representative H&E staining images of adipose tissue (Epi-fat) of BM transplanted mice. (**b**) Left, representative Masson’s trichrome staining images of adipose tissue (Epi-fat) of BM transplanted mice. Right, quantification of Masson’s trichrome-positive area (identical with interstitial fibrosis). n = 3 images per mouse, 5–10 mice each. ****p < 0.0001 by unpaired t-test. (**c**) Left, representative immunofluorescent staining of F4/80 (red) and perilipin (green). Right, the number of F4/80-positive cells in Epi-fat of BM transplanted mice. Scale bars, 100 μm. ****p < 0.0001 by unpaired t-test. (**d**) Collagen (*Col1a1, Col3a1* and *Col6a1*) mRNA levels in Epi-fat from BM transplanted mice. n = 9–15, *p < 0.05 by unpaired t-test. (**e**) Pro-inflammatory (*Emr1*, *Mcp1* and *Tnf-α*) mRNA levels in Epi-fat from BM transplanted mice. n = 9–15, *p < 0.05 by unpaired t-test.

Epi-fat from BM-*Postn*^+/+^ mice was characterized by higher interstitial fibrosis compared with those from BM-*Postn*^−/−^ mice, as determined using Masson’s trichrome staining (Fig. 8a,b). By double-immunofluorescent staining of F4/80 (red) and perilipin (green), we observed that the number of F4/80-positive cells in BM-*Postn*^−/−^ mice was reduced compared with BM-*Postn*^+/+^ mice (Fig. 8c). We also observed higher mRNA expression levels of pro-fibrotic collagen types I, III, and VI (*Col1a1*, *Col3a1*, and *Col6a1*) and inflammatory genes (*Emr1*, *Mcp1*, and *Tnf-α*) in HFD-fed BM-*Postn*^+/+^ mice than in BM-*Postn*^−/−^ mice (Fig. 8d,e). In addition, adipocyte size from the Epi-fat was larger in HFD-fed BM-*Postn*^−/−^ mice than in BM-*Postn*^+/+^ mice (Supplementary Fig. 6d). These results were the same as those of the whole-body *Postn*^−/−^ phenotype.

Finally, we examined the effect of *Postn* deficiency in hematopoietic cells on glucose homeostasis in HFD-fed mice. Fasting glycemia, fasting insulin concentration, and insulin secretion during IPGTTs were not different between BM-*Postn*^+/+^ and BM-*Postn*^−/−^ mice. There was no change between hematopoietic genotypes in either IPGTTs or ITTs (Supplementary Fig. 7a–h).

## Discussion

In this study, we revealed that *Postn* is produced at extraordinary levels from adipose tissue after HFD feeding for the first time. Macrophages in visceral adipose tissue were responsible for the *Postn* production and secretion during the development of obesity, possibly due to hypoxia. *Postn*^−/−^ mice fed HFD had less CLS formation and fibrosis in adipose tissue and were protected from liver steatosis and systemic insulin resistance compared with WT littermates. Mice deficient of *Postn* in their hematopoietic compartment also had lower inflammation in adipose tissue, in parallel with a reduction in ectopic lipid accumulation compared with controls.

Adipose tissue expansion is required to store surplus energy efficiently and prevent ectopic lipid deposition in metabolically sensitive tissues, thus preventing metabolic complications. Acute and local inflammation within adipose tissue plays an essential role in healthy adipose tissue expansion, remodeling, and overall homeostasis by stimulating ECM degradation and angiogenesis^1^. However, during the development of obesity, chronic, low-grade inflammation is induced in adipose tissue^25,26^. Increased adipose tissue inflammation stimulates adipocyte lipolysis and tissue fibrosis, and thus led to increases in metabolic abnormalities, such as type 2 diabetes and coronary artery disease^27^. It has been accepted that abnormal collagen deposition is a key feature of adipose tissue dysfunction during obesity^28^. However, how ECM dynamics reflect states of pathological fibrosis in response to over-nutrition is poorly understood. In this study, we showed that Periostin (*Postn*) is a novel regulator of adipose tissue fibrosis.

*Postn*, which is secreted as a latent consequence of inflammatory responses, sustains or amplifies the inflammation response^13,29^. In fact, *Postn* expression is quite low in adipose tissue in healthy lean mice. We observed that *Postn* expression in Epi-fat changed in parallel with inflammatory gene expression during the development of obesity. It is conceivable that *Postn* is involved in pathological tissue remodeling in adipose tissue. Because *Postn* is required for the regulation of ECM production and maturation, the loss of *Postn* resulted in decreased collagen cross-linking and the degradation of ECM^30^. Targeted deletion of matrix-involved protein such as collagen VI or matrix metallopeptidase 12 in obese mice resulted in the healthy expansion of individual adipocytes^31,32^. In contrast, abnormal collagen accumulation in association with developmental fibrosis limited adipose tissue expansion^33,34^. Consistent with these studies, we observed that systemic *Postn* deficiency resulted not only in reduced accumulation of macrophages and abnormal collagen deposition but also in larger adipocyte cell size in Epi-fat. At the same time, Epi-fat weight increase with a reciprocal reduction in liver weight was observed in *Postn*^−/−^ mice, which indicated that the adipose tissue fibrosis induced by Postn is a detrimental factor for ectopic lipid disposition and insulin resistance in obesity. A recent study demonstrated that aberrant expression of *Postn* in the liver resulted in steatosis and hypertriglyceridemia through JNK-mediated suppression of fatty acid oxidation^20^. However, the induction of Postn in adipose tissue was approximately 80 times higher than those observed in the liver in our experiment, suggesting its potent role in metabolic abnormalities in the obese condition.

ECM dynamics are believed to occur in the pro-inflammatory context of hypertrophic adipocyte, local hypoxia, and pathological immunity. Macrophages, which are known to drive an inflammatory program within obese adipose tissue, are believed to also orchestrate fibrogenesis in several organs^35,36^. Inflammatory macrophages accumulated in the visceral adipose tissue of obese mice, especially in hypoxic areas. The reasons included: (1) macrophages act to remove dead adipocytes; and (2) macrophages are trapped in hypoxic areas by macrophage migration inhibition factor^37^. Of note, *Postn* was reported to be increased in hypoxic conditions in non-small cell lung cancer^38^. In addition, a previous study showed that *Postn* is secreted from infiltrated inflammatory cells and myofibroblasts in valvular heart disease^39^. Here, we demonstrated that macrophages, both J774.1 cells and peritoneal macrophages, exposed to hypoxia secreted *Postn*. *Postn* expression were remarkably increased in F4/80^+^ cells from Epi-fat in HFD-fed mice, but it was rarely expressed in SD-fed mice or adipocytes. It is tempting to speculate that adipose tissue macrophages in obese Epi-fat were recruited by hypoxia-inducible *Postn*. Consistent with this, previous studies have shown that hypoxia increased *Postn* expression and promoted the recruitment of monocyte-derived macrophages from peripheral blood through the integrin α_v_β3 in a mouse model of glioblastoma^30^.

*Postn* is expressed both in infiltrated macrophages and other fibroblast like cells in adipose tissue. However, the contribution of macrophages is poorly understood. To address this question, we used BM transplantation to generate chimeric mice with knockout of *Postn* specifically in hematopoietic cells. Our results demonstrated that *Postn* deficiency in hematopoietic cells suppressed *Postn* expression in Epi-fat and decreased macrophage infiltration and fibrosis after HFD feeding, which was accompanied by a reduction in ectopic lipid accumulation in the liver. These findings were compatible with the whole-body *Postn*^−/−^ phenotypes. However, we could not observe amelioration of insulin resistance in BM-*Postn*^−/−^ mice compared with WT littermates. This may be because of the low increase in body weight in this experiment, possibly caused by the effect of whole-body irradiation.

Our studies demonstrated that the F4/80^−^ SVF also secreted Postn after HFD feeding. A recent study showed that numbers of αSMA-positive cells or myofibroblasts existed in obese adipose tissue^10^. Both myofibroblast marker (*αSMA*) and preadipocyte marker (*Pdgfr-α* and *Ppar-γ*) levels were increased in the F4/80^−^ SVF from obese Epi-fat compared with those from lean mice, suggesting that *αSMA*-positive cells may contribute to the expression of *Postn* in the F4/80^−^ SVF in adipose tissue (Supplementary Fig. 4b).

*Postn*^−/−^ mice tended to have lower body weight than littermate controls. As reported, *Postn*^−/−^ mice showed reduced body weight compared with WT littermates after weaning because of problems with their teeth^40,41^. In our case, there was no difference in HFD intake (Supplementary Fig. 2a) possibly because HFD is soft. As a result, there was no difference in body weight between genotypes when IPGTTs and ITTs were conducted. Moreover, our chimeric mice had *Postn* deficiency only in bone marrow-derived hematopoietic cells and there was no difference in body weight between chimeric and control mice. Thus, the phenotypic differences observed in our experiments were due entirely to the presence or absence of *Postn*.

In summary, we demonstrated that *Postn* has a critical role in HFD-induced adipose tissue inflammation and fibrosis, which may reduce lipid storage capacity and enhance ectopic lipid accumulation. Our data indicated that the regulation of *Postn* in visceral fat could be beneficial for the maintenance of healthy adipose tissue in obese patients (Supplementary Fig. 8).

## Methods

All data generated or analysed during this study are included in this published article (and its Supplementary Information files).

### Animals

Periostin (*Postn*) whole-body knockout mice were generated by Dr. Kudo^42^. We crossed these mice and C57BL/6J mice (purchased from Japan SLC) to generate the *Postn*^−/−^ and *Postn*^+/+^ littermate controls in a C57BL/6J genetic background. HFD (Research Diet, 60% fat) was started at 8 weeks of age and continued for 16 weeks with ad libitum access to food. We generated *Rosa26-loxP-stop-loxP-tdTomato* transgenic mice by crossing with *Periostin-Cre* mice, in which *tdTomato* activates the Postn-specific promoter to induce *cre* expression. *Periostin-Cre* mice (generated by Dr. Conway and colleagues^43,44^) were provided by Dr. Manabe (Chiba University). *tdTomato* mice were obtained from The Jackson Laboratory. Mice were maintained in specific pathogen-free conditions at the Institute of Laboratory Animals of Kyoto University Graduate School of Medicine. This study was approved by the Kyoto University Ethics Review Board. All methods were performed in accordance with the relevant guidelines and regulations.

### Histological analysis

Adipose fat tissues and liver were fixed in 4% paraformaldehyde and embedded in paraffin. Areas of fibrosis were measured using image analysis software (Axio Observer 7, ZEISS). CLSs in Epi-fat were detected immunohistochemically using an anti-F4/80 antibody (sc-59171, Santa Cruz Biotechnology) and an anti-perilipin antibody (NB100-60554, Novus Biologicals). The CLS density was obtained from the number of CLSs per 1,000 adipocytes in each section^45^. They were counted in more than 5 mm^2^ areas of each section per mouse, and the number of CLSs was expressed as the mean number per mm^2^.

### Confocal microscopic analysis

For confocal microscopic analysis, the adipose tissues from fluorescent reporter mice stained with fluorescent antibodies were visualized in whole mounts, as described previously^46^. In brief, Epi-fat cut into small pieces was fixed 1% paraformaldehyde for 30 min and then permeabilized with 0.5% Triton X-100 prior to staining. The fluorescent stains were examined using an SP8 confocal microscope system (Leica).

### Western blotting

Western blotting was performed using standard procedures as described previously^47^. Samples were lysed in lysis buffer consisting of 100 mM Tris-HCl, pH 7.4, 75 mM NaCl, and 1% Triton X-100 (Nacalai Tesque). The lysis buffer was supplemented with complete mini protease inhibitor (Roche), 0.5 mM NaF and 10 mM Na_3_VO_4_ just before use. The protein concentration was determined using a bicinchoninic acid (BCA) protein assay kit (Bio-Rad). All samples (10 μg of protein) were suspended in lysis buffer, fractionated using NuPAGE 4–12% Bis-Tris (Invitrogen) gels, and transferred to a Protran nitrocellulose transfer membrane (Whatman). The membrane was blocked using 1× phosphate-buffered saline (PBS) containing 5% non-fat milk for 1 h and incubated with the primary antibodies against Periostin/OSF-2 (NBP1-30042, Novus Biologicals), HIF-1 (NB100-479, Novus Biologicals), or GAPDH (14C10, cell signaling) overnight at 4 °C. After a washing step in PBS with 0.05% Tween 20 (0.05% T-PBS), the membrane was incubated with a secondary antibody (anti-rabbit, anti-mouse, or anti-goat IgG horseradish peroxidase (HRP)-linked; 1:2,000) for 1 hour at room temperature. The membrane was then washed in 0.05% T-PBS, and signals were detected using ECL Western Blotting Detection Reagent (GE Healthcare) with an LAS-4000 system (GE Healthcare Life Science).

### Western blotting for AKT *in vivo*

After an 18-h fast, mice were injected with insulin (1 U kg^−1^) or saline via the inferior vena cava. Liver and Epi-fat were removed 1 and 3 min after the injection, respectively. The following antibodies were used: anti-phospho-AKT (Thr308) (#4056, Cell Signaling) and anti-AKT (#9227, Cell Signaling).

### Triglyceride content in the liver

Livers were homogenized in ice-cold chloroform/methanol (2:1 v/v). Lipid extracts were prepared by the Folch method^48^. Triglyceride content in the liver was measured using a Triglyceride E-test Wako (Wako Pure Chemical Industries).

### Glucose and insulin tolerance tests

For GTTs, after overnight fasting, male mice, after feeding with SD or HFD for 12 weeks and HFD after transplantation for 16 weeks, were injected with 1.0 g kg^−1^ glucose intraperitoneally. For ITTs, after a 4-h fast, male mice at 10 and 16 or 20 weeks of age were injected intraperitoneally with insulin (1 U kg^−1^ for SD-fed and HFD-fed mice after BM transplantation, and 2.0 U kg^−1^ for HFD-fed mice; Humulin R; Eli Lilly Japan KK). Blood was obtained from the orbital vein, and glucose levels were measured using a glucose sensor.

### Measurement of serum insulin levels

We quantified serum levels of insulin in male mice after 14 weeks of SD or HFD feeding and after BM transplantation at 18 weeks for HFD feeding using an ELISA assay kit for mouse insulin in accordance with the manufacturer’s instructions (Shigayagi Co. Ltd, Shibukawa, Japan).

### Biochemical analysis of serum

After mice were fasted for 4–6 h, blood was obtained from the inferior vena cava of anaesthetized mice, and serum was separated by centrifugation at 4 °C and stored at −80 °C. Biochemical data were measured by standard methods using a Hitachi 7180 Auto Analyzer (Nagahama Life Science Laboratory, Nagahama, Japan).

### Stromal vascular fractionation and flow cytometry analysis

We isolated adipocytes and stromal vascular cells as described previously^49,50^. In brief, we removed Epi-fat tissue and minced it into small pieces in Krebs Ringer phosphate buffer and stirred using a shaker at 100 rpm for 20–30 min in collagenase solution with 3 mg mL^−1^ collagenase type 2 (Worthington). After filtering thought nylon mesh, we centrifuged at 300 × *g* for 10 min and collected the floating adipocyte layer. We resuspended the resultant pellet containing stromal vascular cells and filtered it through a 70-μm mesh. We washed the stromal vascular cells twice with PBS, and finally resuspended them in PBS supplemented with 2% fetal bovine serum (FBS). We added 50 ng of Fcblock antibody per 1 × 10^6^ cells and incubated these isolated cells with either labeled anti-F4/80 (#123107, Biolegend) antibody or isotype control antibody (#400110, Biolegend) and analyzed by flow cytometry using a FACSAria II (BD Biosciences) and FlowJo software (Tomy Digital Biology).

### Cell culture and Transwell assays

Macrophage cell line J774.1 were seeded at a density of 10,000 cells/mL in the upper chamber and then allowed to migrate for 48 h before fixation for Geimsa staining. Migration assays were performed in media with 10% FBS or the media with 0.1% bovine serum albumin with Recombinant human POSTN protein (R&D system) 0.05 ug/ml and 0.5 ug/ml or vesicles were measured in the lower chamber. Peritoneal macrophages were obtained from the peritoneal cavity of *Postn*^+/+^ or *Postn*^−/−^ mice 4 days after intraperitoneal injection of 3 mL of 3% thioglycollate. The cells obtained were washed, spun at 1,000 rpm for 5 min. Transwell assays were performed on 24-well plates with inserts (Millicell). *Postn*^+/+^ or *Postn*^−/−^ peritoneal macrophages were seeded at a density of 10,000 cells/mL in the upper chamber and then allowed to migrate for 48 h before fixation for Geimsa staining. Migration assays were performed in media with 10% FBS or the media with 0.1% bovine serum albumin with TGFβ (R&D system), Recombinant human POSTN protein (R&D system) or vesicles were measured in the lower chamber.

### Bone marrow transplantation

BM transplantation was conducted as described previously^51,52^. Eight-week-old female mice with genotypes of *Postn*^+/+^ and *Postn*^−/−^ were used as BM donors. BM recipients were 8 weeks old male B57BL/6J (Japan SLC). BM donors were euthanized by cervical dislocation, and BM cells were collected by flushing femurs and tibias with PBS supplemented with 2% FBS. The suspension was passed through 40-μm mesh. Red blood cells were lysed using ACK lysing buffer (Lonza). BM cells were then washed twice with PBS supplemented with 2% FBS. Recipients were irradiated with two doses of 6 Gy within an interval of 3 hours (cesium 137; Gammacell 40 Exactor) and injected intravenously with 2 × 10^6^ BM cells 6 hours after irradiation. Transplanted mice were housed in microisolator housing for 4 weeks prior to challenge with an HFD.

### RNA extraction and qRT-PCR

Total RNA was isolated and purified using TriPure Isolation Reagent (Roche), and cDNA was synthesized from 100 ng of total RNA using a Transcriptor First Strand cDNA Synthesis Kit (Roche) in accordance with the manufacturer’s instructions. For quantitative RT-PCR, specific genes were amplified by 40 cycles using SYBR^TM^ Green PCR Master Mix (Applied Biosystems). Expression was normalized to the 18S ribosomal RNA housekeeping gene. Gene-specific primers are listed in Supplementary Table S1.

### Statistical analysis

Data are presented as means ± standard error of the mean (SEM). Statistical comparisons were performed using unpaired t-test or one-way analysis of variance (ANOVA) with Sidak’s post-hoc test (three or more groups) were used as indicated in the figure legends. A p value of <0.05 was considered as statistically significant. Statistical analyses were performed using GraphPad Prism 6 (GraphPad Software, Inc.).

## Electronic supplementary material

Supplementary information

## References

[1] Halberg N (2009). Hypoxia-inducible factor 1 induces fibrosis and insulin resistance in white adipose tissue. Mol. Cell. Biol..

[2] Lawrence T, Natoli G (2011). Transcriptional regulation of macrophage polarization: enabling diversity with identity. Nat. Rev. Immunol..

[3] Odegaard JI, Chawla A (2013). Pleiotropic actions of insulin resistance and inflammation in metabolic homeostasis. Science..

[4] Liao X (2011). Kruppel-like factor 4 regulates macrophage polarization. J. Clin. Invest..

[5] Odegaard JI (2007). Macrophage-specific PPAR- controls alternative activation and improves insulin resistance. Nature.

[6] Suganami T, Tanaka M, Ogawa Y (2012). Adipose tissue inflammation and ectopic lipid accumulation. Endocr. J..

[7] Sun K, Kusminski CM, Scherer PE (2011). Adipose tissue remodeling and obesity. J. Clin. Invest..

[8] Jonker JW (2012). A PPARγ-FGF1 axis is required for adaptive adipose remodelling and metabolic homeostasis. Nature..

[9] Sun K, Halberg N, Khan M, Magalang UJ, Scherer PE (2013). Selective  inhibition of hypoxia-inducible factor 1a ameliorates adipose tissue dysfunction. Mol. Cell. Biol..

[10] Miyako T (2014). Macrophage-inducible C-type lectin underlies obesity-induced adipose tissue fibrosis. Nature com..

[11] Zinn K, McAllister L, Goodman CS (1988). Sequence analysis and neuronal expression of fasciclin I in grasshopper and Drosophila. Cell..

[12] Horiuchi K (1999). Identification and characterization of a novel protein, periostin, with restricted expression to periosteum and periodontal ligament and increased expression by transforming growth factor beta. J Bone Miner Res..

[13] Nishiyama T (2011). Delayed re-epithelialization in periostin-deficient mice during cutaneous wound healing. PLoS One..

[14] Zhou W (2015). Periostin secreted by glioblastoma stem cells recruits M2 tumour-associated macrophages and promotes malignant growth. Nat Cell Biol..

[15] Xiaofan G (2016). Hypoxia promotes glioma-associated macrophage infiltration via peristin and subsequent M2 polarization by upregulating TGF-beta and M-CSFR. Oncotarget..

[16] Norris RA (2007). Periostin regulates collagen fibrillogenesis and the biomechanical properties of connective tissues. J Cell Biochem..

[17] Snider P (2008). Periostin is required for maturation and extracellular matrix stabilization of noncardiomyocyte lineages of the heart. Circ Res..

[18] Oka T (2007). Genetic manipulation of periostin expression reveals a role in cardiac hypertrophy and ventricular remodeling. Circ Res..

[19] Liu AY (2014). Periostin, a multifunctional metricellular protein in inflammatory and tumor microenvironments. Matrix Biol..

[20] Yan L (2014). Periostin promotes liver steatosis and hypertriglyceridemia thorough downregulation of PPARα. J Clin Invest..

[21] Simon H (2016). Loss of periostin results in impaired regeneration and pancreatic atrophy after cerulean-induced pancreatitis. Am. J. Pathol..

[22] Ye J (2007). Hypoxia is a potential risk factor for chronic inflammation and adiponectin reduction in adipose tissue of ob/ob and dietary obese mice. Am. J. Physiol. Endocrinol. Metab..

[23] Hosogai N (2007). Adipose tissue hypoxia in obesity and its impact on adipocytokine dysregulation. Diabetes..

[24] Rausch ME (2008). Obesity in C57BL/6J mice is characterized by adipose tissue hypoxia and cytotoxic T-cell infiltration. Int. J. Obes..

[25] Hotamisligil GS (2006). Inflammation and metabolic disorders. Nature..

[26] Olefsky JM, Glass CK (2010). Macrophages, inflammation, and insulin resistance. Annu. Rev. Physiol..

[27] Park J (2011). Paracrine and endocrine effects of adipose tissue on cancer development and progression. Endocr. Rev..

[28] Sun K (2013). Fibrosis and adipose tissue dysfunction. Cell Metab..

[29] Matsuoka M (2012). Postn promotes chronic allergic inflammation in response to Th2 cytokines. J. Clin Invest..

[30] Jennifer A (2016). Deletion of periostin protects against atherosclerosis in mice by altering inflammation and extracellular matrix remodeling. Arterioscler Thromb Vasc Biol..

[31] Khan T (2009). Metabolic dysregulation and adipose tissue fibrosis: role of collagen VI. Mol. Cell. Biol..

[32] Lee JT (2014). Macrophage metalloelastase (MMP12) regulates adipose tissue expansion, insulin sensitivity, and expression of inducible nitric oxide synthase. Endocrinology.

[33] Chun TH (2006). A pericellular collagenase directs the 3-dimensional development of white adipose tissue. Cell.

[34] Vila IK (2014). Immune cell toll-like receptor 4 mediates the development of obesity- and endotoxemia-associated adipose tissue fibrosis. Cell Rep..

[35] Wynn TA, Barron L (2010). Macrophages: master regulators of inflammation and fibrosis. Semin. Liver Dis..

[36] Song E (2000). Influence of alternatively and classically activated macrophages on fibrogenic activities of human fibroblasts. Cell. Immunol..

[37] Rausch ME (2008). Obesity in C57BL/6J mice is characterized by adioise tissue hypoxia and cytotoxic T-cell infiltration. Int. J. Obes..

[38] Ouyang G (2009). Upregulated expression of periostin by hypoxia in non- small-cell lung cancer cells promotes cell survival via the Akt/PKB pathway. Cancer Lett..

[39] Hakuno D (2010). Periostin advances atherosclerotic and rheumatic cardiac valve degeneration by inducing angiogenesis and MMP production in humans and rodents. J Clin Invest..

[40] Maude G (2015). Periostin expression contributes to cortical bone loss during unloading. Bone..

[41] Kruzynska-Frejtag A (2004). Periostin is expressed within the developing teeth at the sites of epithelial-mesenchymal interaction. Dev Dyn..

[42] Shimazaki M (2008). Impaired capsule formation of tumors in periostin null mice. Biochem Biophys Res Commun..

[43] Takeda N (2010). Cardiac fibroblasts are essential for the adaptive response of the murine heart to pressure overload. J Clin Invest..

[44] Lindsley A (2007). Identification and characterization of a novel Schwann and outflow tract endocardial cushion lineage-restricted periostin enhancer. Dev Biol..

[45] Murano I (2008). Dead adipocytes, detected as crown-like structures, are prevalent in visceral fat depots of genetically obese mice. J. Lipid. Res..

[46] Ryan B (2014). Imaging of adipose tissue. Methods in Enzymology.

[47] Horie T (2010). Acute doxorubicin cardiotoxicity is associated with miR-146a- induced inhibition of the neuregulin-ErbB pathway. Cardiovasc. Res..

[48] Folch J (1957). A simple method for the isolation and purification of total lipides from animal tissues. J. Biol. Chem..

[49] Nishimura S (2013). Adipose natural regulatory B cells negatively control adipose tissue inflammation. Cell Metab..

[50] Bolton K (2008). Decorin is a secreted protein associated with obesity and type 2 diabetes. Int J Obes..

[51] Gokani VJ (2015). A retrospective study: factors associated with the risk of abdominal aortic aneurysm rupture. Vascul Pharmacol..

[52] Rayner KJ (2011). Antagonism of miR-33 in mice promotes reverse cholesterol transport and regression of atherosclerosis. J Clin Invest..

