# Supplementary material for: Loss of periostin ameliorates adipose tissue inflammation and fibrosis in vivo
Source: Sci Rep. 2018 Jun 4;8:8553. doi: 10.1038/s41598-018-27009-9 (PMC5986813; doi:10.1038/s41598-018-27009-9)
Supplement: Supplementary file 1 — Supplementary information [file 41598_2018_27009_MOESM1_ESM.pdf]

Loss of periostin ameliorates adipose tissue inflammation and fibrosis *in vivo*

Fumiko Nakazeki[1], Masataka Nishiga[1], Takahiro Horie[1], Hitoo Nishi[1], Yasuhiro Nakashima[1], Osamu Baba[1], Yasuhide Kuwabara[1], Tomohiro Nishino[1], Tetsushi Nakao[1], Yuya Ide[1], Satoshi Koyama[1], Masahiro Kimura[1], Shuhei Tsuji[1], Naoya Sowa[1], Shigeo Yoshida[2], Simon J Conway[3], Motoko Yanagita[4], Takeshi Kimura[1], and Koh Ono[1]

[1]Department of Cardiovascular Medicine, Graduate School of Medicine, Kyoto University, Kyoto 606-8507, Japan
[2]Department of Ophthalmology, Kyushu University Graduate School of Medical Sciences, Fukuoka 812-8582, Japan
[3]Herman B Wells Center for Pediatric Research, Indiana University of Medicine, Indianapolis, Indiana, USA
[4]Department of Nephrology, Graduate School of Medicine, Kyoto University, Kyoto 606-8507, Japan

Supplementary Table S1

Used gene-specific oligonucleotide primer sequences

Gene	Forward	Reverse
Postn	AACCAAGGACCTGAAACACG	GTGTCAGGACACGGTCAATG
Emr1	TCCAGAAGGCTCCCAAGGATA	GGGCACTTTTGTTCTCACAGGTA
Mcp1	AGGTCCCTGTCATGCTTCTG	GCTGCTGGTGATCCTCTTGT
Tnf-α	CCAGACCCTCACACTCAGATC	CACTTGGTGGTTTGCTACGAC
Col1a1	GCCAAGAAGACATCCCTGAAG	TCATTGCATTGCACGTCATC
Col3a1	ACCAAAAGGTGATGCTGGAC	GACCTCGTGCTCCAGTTAGC
Col6a1	TGCAGGCATTGAGATCTTTG	CAGGGCCTGGTAGTTTGGTA
Pgk1	TGTCGCTTTCCAACAAGCTG	GGTGGCTCATAAGGACAACG
Glut1	GGGCATGTGCTTCCAGTATGT	ACGAGGAGCACCGTGAAGAT
Vimentin	AGATCGATGTGGACGTTTCC	TCCGGTACTCGTTTGACTCC
α-Sma	TCCCTGGAGAAGAGCTACGAACT	GATGCCCGCTGACTCCAT
Pdgfr-α	TCCTTCTACCACCTCAGCGAG	CCGGATGGTCACTCTTTAGGAAG
C/Ebp-α	CTGCGAGCACGAGACGTCTA	CTGTCGGCTGTGCTGGAA
Vegf-α	CTGCCGTCCGATTGAGACC	CCCCTCCTTGTACCACTGTC
18S RNA	CGCGGTTCTATTTTGTTGGT	AGTCGGCATCGTTTATGGTC

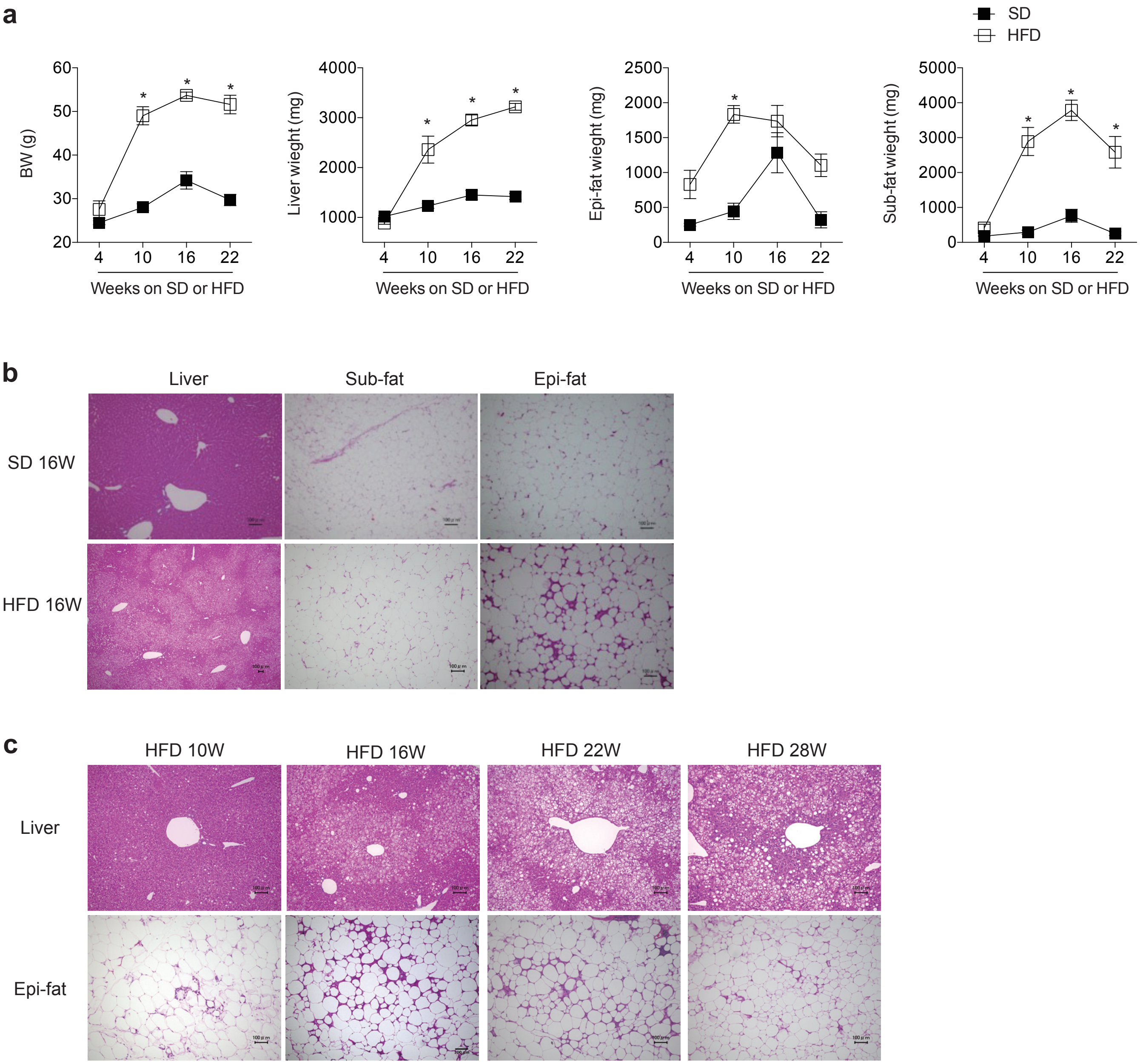

Supplementary Fig. 1. Phenotypes of SD-fed or HFD-fed WT mice.

(**a**) Time course of body, liver, and adipose tissue weights of WT mice after SD or HFD feeding for up to 30 weeks. n=3-6, *P<0.05, **P<0.01, ***P<0.001, ****P<0.0001 versus SD feeding at each time point. Statistical comparisons were made by unpaired t-test. (**b**) Representative H&E staining images of tissues of WT mice after SD or HFD feeding for 16 weeks. Scale bars, 100μm. (**c**) Representative H&E staining images of tissues of WT mice after HFD feeding for 10, 16, 22 and 28 weeks. Scale bars, 100μm.

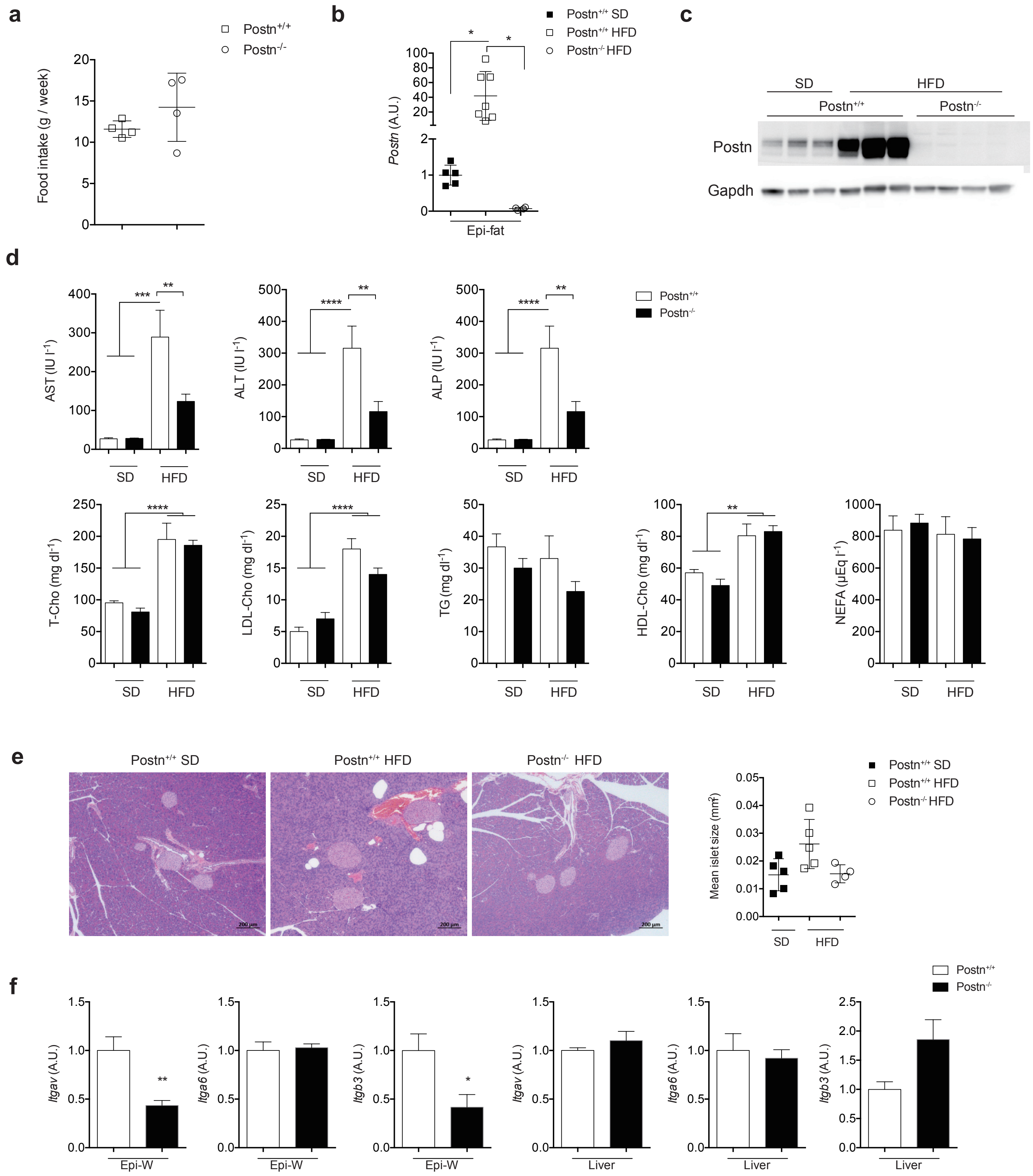

Supplementary Fig. 2. Analysis of *Postn*$^{+/+}$ and *Postn*$^{-/-}$ mice fed a SD or a HFD.

(**a**) Food intake of *Postn*$^{+/+}$ and *Postn*$^{-/-}$ mice fed HFD in metablic cages. n=4 each, by unpaired t-test. (**b**) mRNA levels of *Postn* in adipose tissue (Epi-fat) from *Postn*$^{+/+}$ and *Postn*$^{-/-}$ mice. n=5-7, *P<0.05 by one-way ANOVA. (**c**) Protein levels of Postn in adipose tissue (Epi-fat) from *Postn*$^{+/+}$ and *Postn*$^{-/-}$ mice. (**d**) Serum profile of *Postn*$^{+/+}$ and *Postn*$^{-/-}$ mice. n=3 for the SD groups, n=8-10 for the HFD groups, **P<0.01, ***P<0.001, ****P<0.0001 by one-way ANOVA. (**e**) Left, representative microscope images of pancreas from *Postn*$^{+/+}$ and *Postn*$^{-/-}$ mice. Right, the mean islet size in pancreas of *Postn*$^{+/+}$ and *Postn*$^{-/-}$ mice. n=4-5, by one-way ANOVA. (**f**) mRNA levels of *Itg* in adipose tissue (Epi-fat) and liver from *Postn*$^{+/+}$ and *Postn*$^{-/-}$ mice. n=5-7, *P<0.05, **P<0.01 by unpaired t-test.

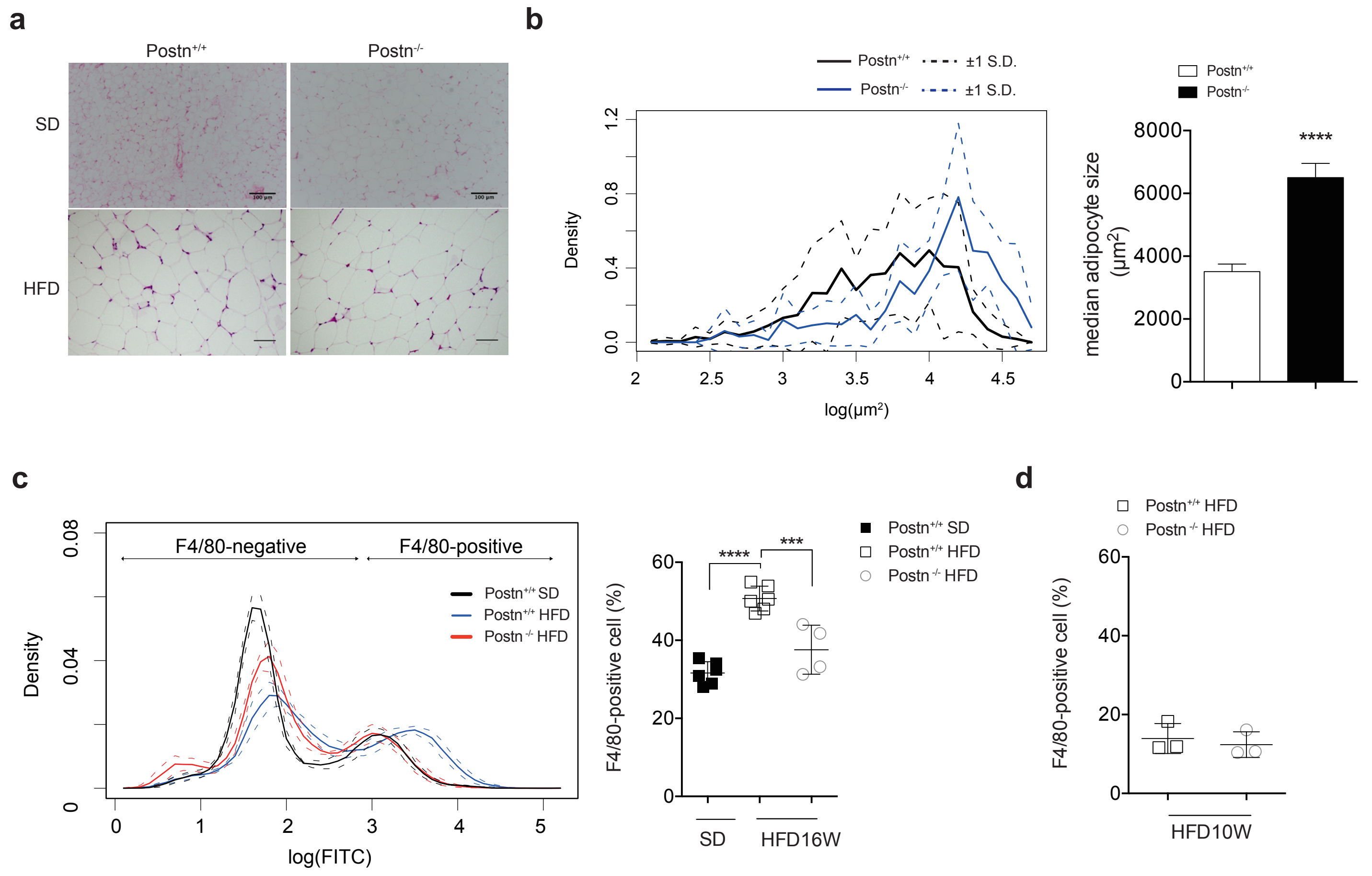

Supplementary Fig. 3. Analysis of the adipose tissues from *Postn*$^{+/+}$ and *Postn*$^{-/-}$ mice.

(**a**) Representative H&E staining images of the adipose tissue (Sub-fat) of SD-fed and HFD-fed *Postn*$^{+/+}$ and *Postn*$^{-/-}$ mice. (**b**) Left, density curve of adipocyte surface areas in Sub-fat. Right, the medium of adipocyte cell size in Sub-fat of *Postn*$^{+/+}$ and *Postn*$^{-/-}$ HFD-fed mice. ***p<0.001 by unpaired t-test. n=3 images per mouse, 10 mice each. (**c**) Left, representative flow cytometry plots showing density curves of F4/80^{+}cells isolated from the Epi-fat. Right, quantification of stromal F4/80^{+}cells isolated from Epi-fat of SD-fed and HFD-fed *Postn*$^{+/+}$ mice and HFD-fed *Postn*$^{-/-}$ mice after 16weeks. n=4 to 6, ***P<0.001, ****P<0.0001 by one-way ANOVA. (**d**) Quantification of stromal F4/80^{+}cells isolated from Epi-fat of HFD-fed *Postn*$^{+/+}$ and *Postn*$^{-/-}$ mice after 10 weeks. n=3 each, by unpaired t-test.

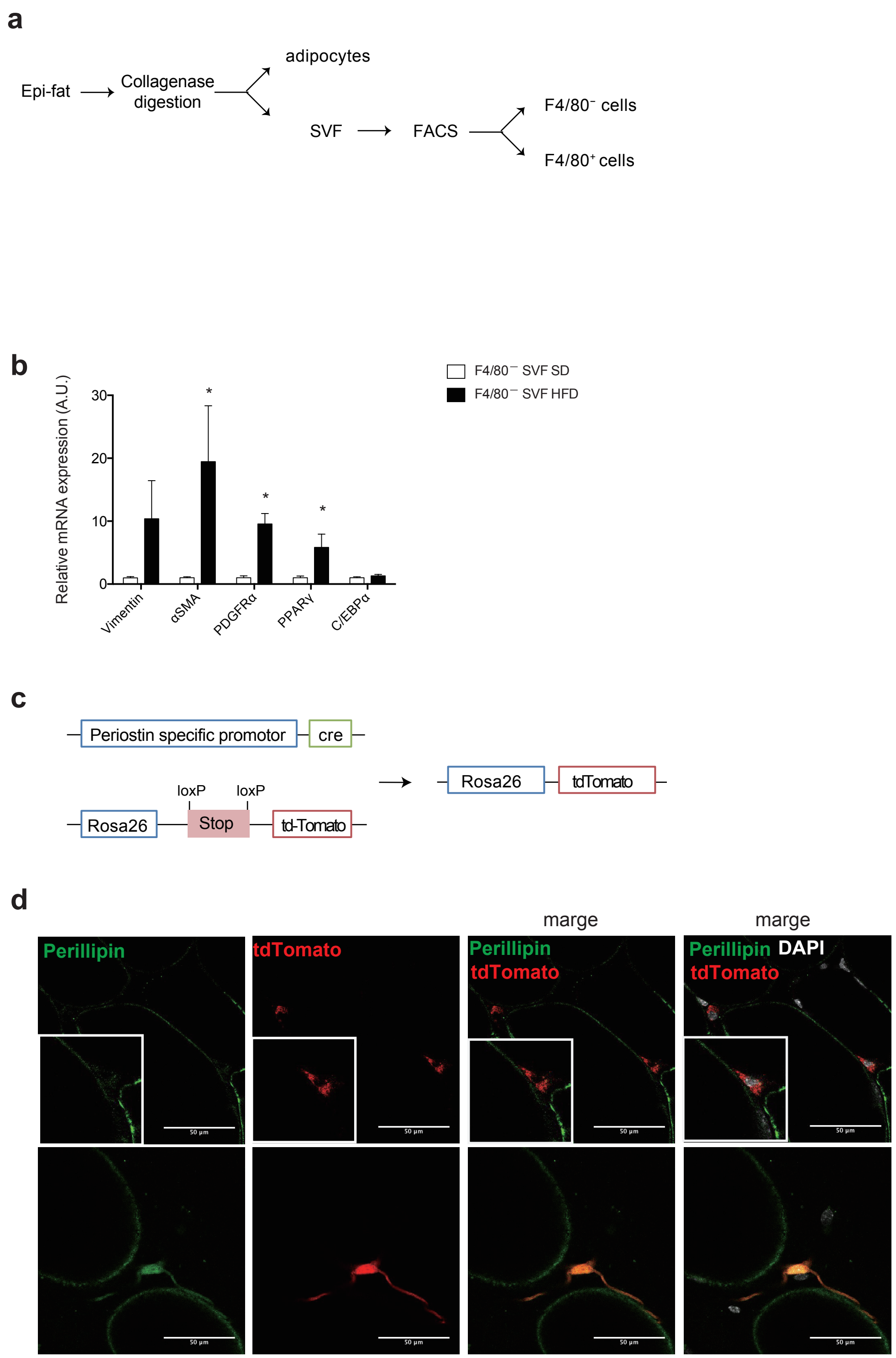

Supplementary Fig. 4. Results of fluorescence-activated cell sorting (FACS) analysis from the SVF.

(**a**) Illustration of the FACS experiments using SVF. (**b**) mRNA expression of *Vimentin, αSMA, Pdgfrα, Ppar-γ* and *C/EBPα* in F4/80^{-} cells. n=5, *p<0.05 unpaired t-test. (**c**) Labeling of *Postn*-expressing cells, generated by crossing *Periostin-Cre* with *Rosa26-loxP-stop-loxP-tdTomato* transgenic mice. (**d**) Representative immunofluorescent staining of perilipin (green) in Epi-fat from obese tdTomato fluorescent protein reporter mice demonstrating Postn-expressing cells. Scar bars, 50μ m.

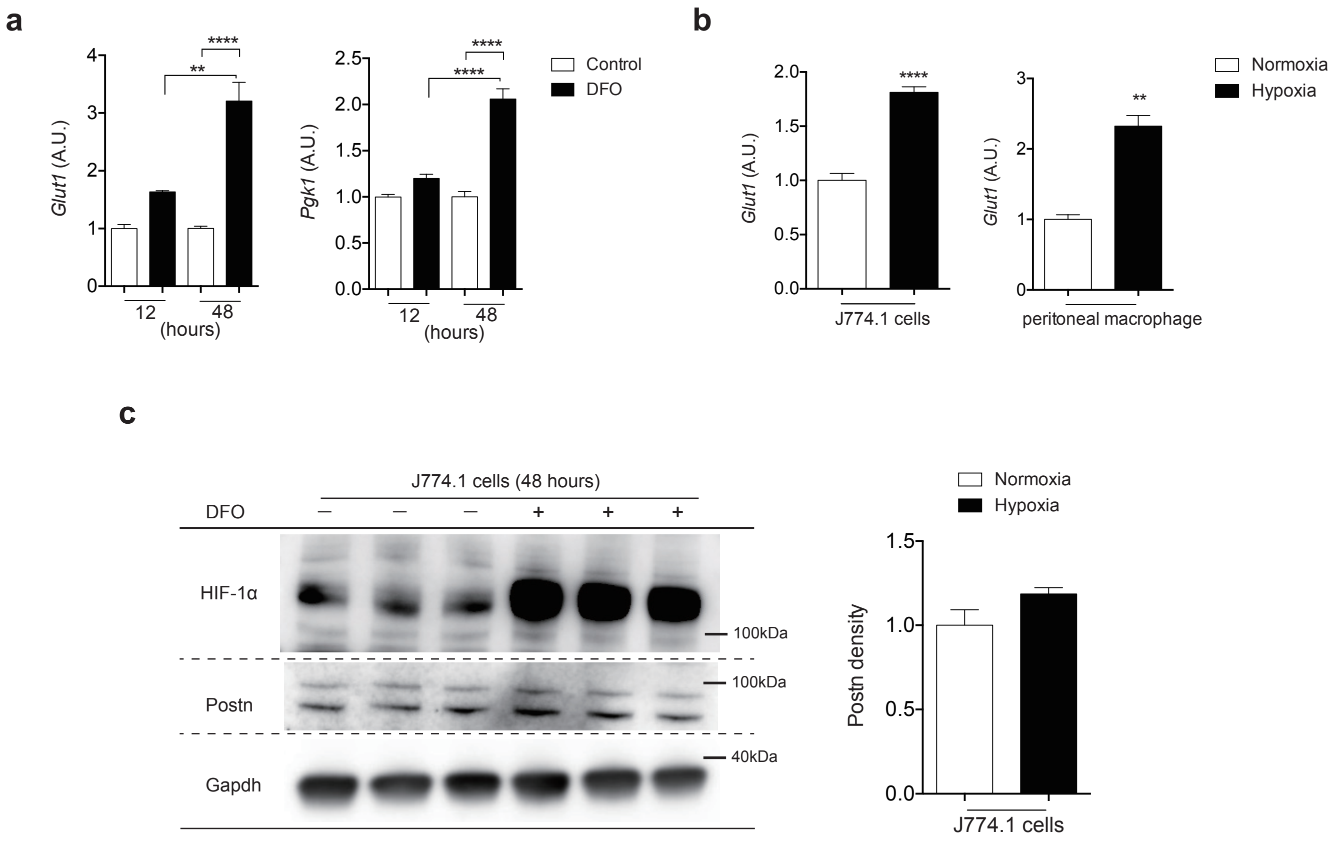

Supplementary Fig. 5. Postn expression under hypoxic condition

(**a**) mRNA expression of the HIF target genes (*Glut1* and *Pgk1*) in J774.1 cells with or without deferoxamine mesylate (DFO) treatment over the indicated time course. n=6, *p<0.05 by one-way ANOVA. (**b**) mRNA expression of *Glut1* in J774.1 cells and peritoneal macrophages exposed hypoxia. n=5 for J774.1, n=5-7 for peritoneal macrophages, *p<0.05 by unpaired t-test. (**c**) Protein levels of Hif-1α and Postn in J774.1 cells with or without DFO treatments. n=3 each, analyzed by one-way ANOVA.

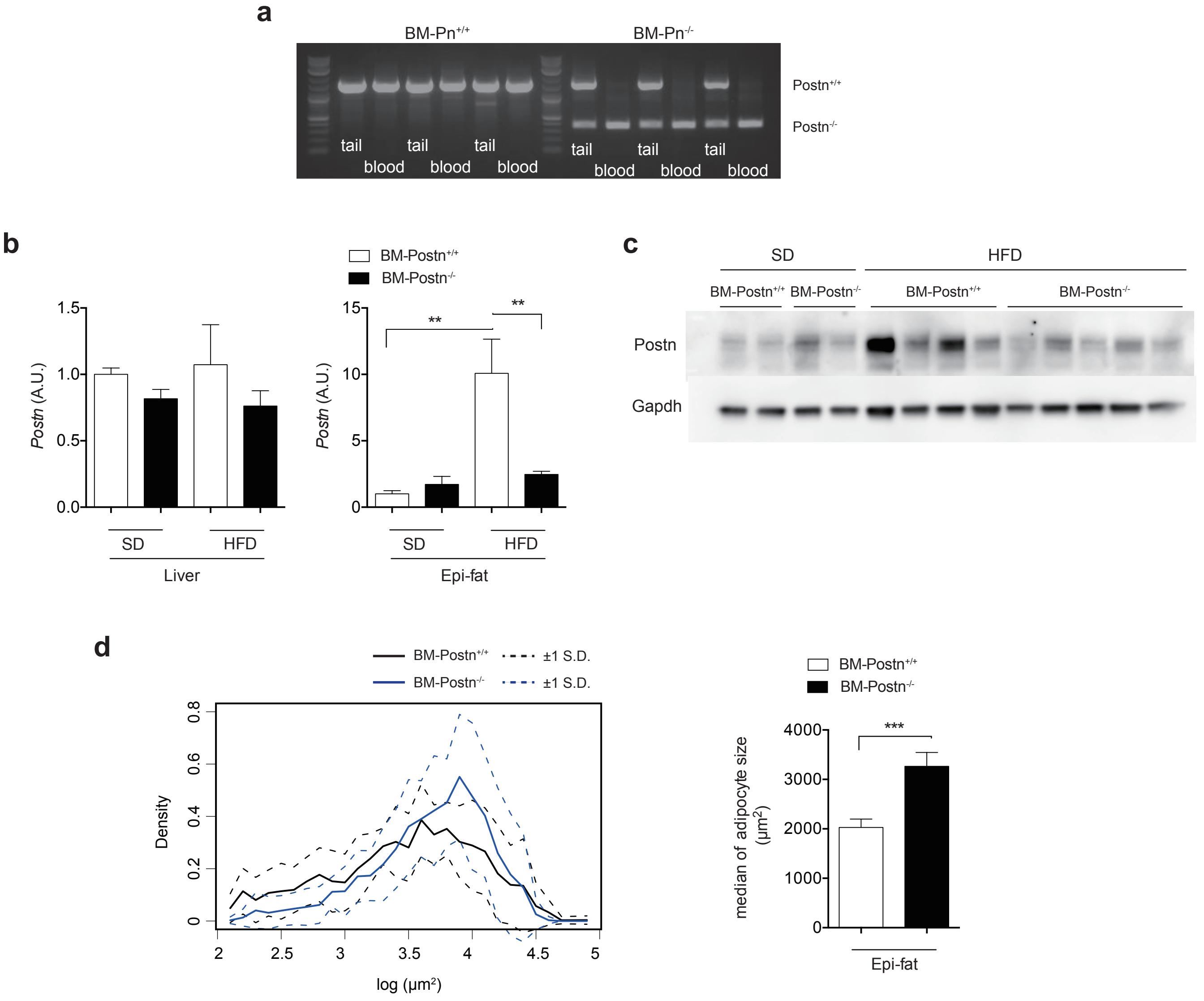

Supplementary Fig. 6. Phenotype of BM transplanted mice.

(**a**) Gel electrophoresis analysis for the *Postn* genomic locus in tail tissue and peripheral blood cells from BM transplanted mice. (**b**) mRNA expression of *Postn* in the liver and Epi-fat from BM transplanted mice. n=11-15, **$p<0.01$ by one-way ANOVA. (**c**) Protein levels of Postn in Epi-fat of BM transplanted mice. (**d**) Left, density curve of adipocyte surface areas. Right, the medium of adipocyte cell size in Epi-fat of HFD-fed, BM transplanted mice. n=3 images per mouse, 9 mice each, ***$p<0.001$ by one-way ANOVA.

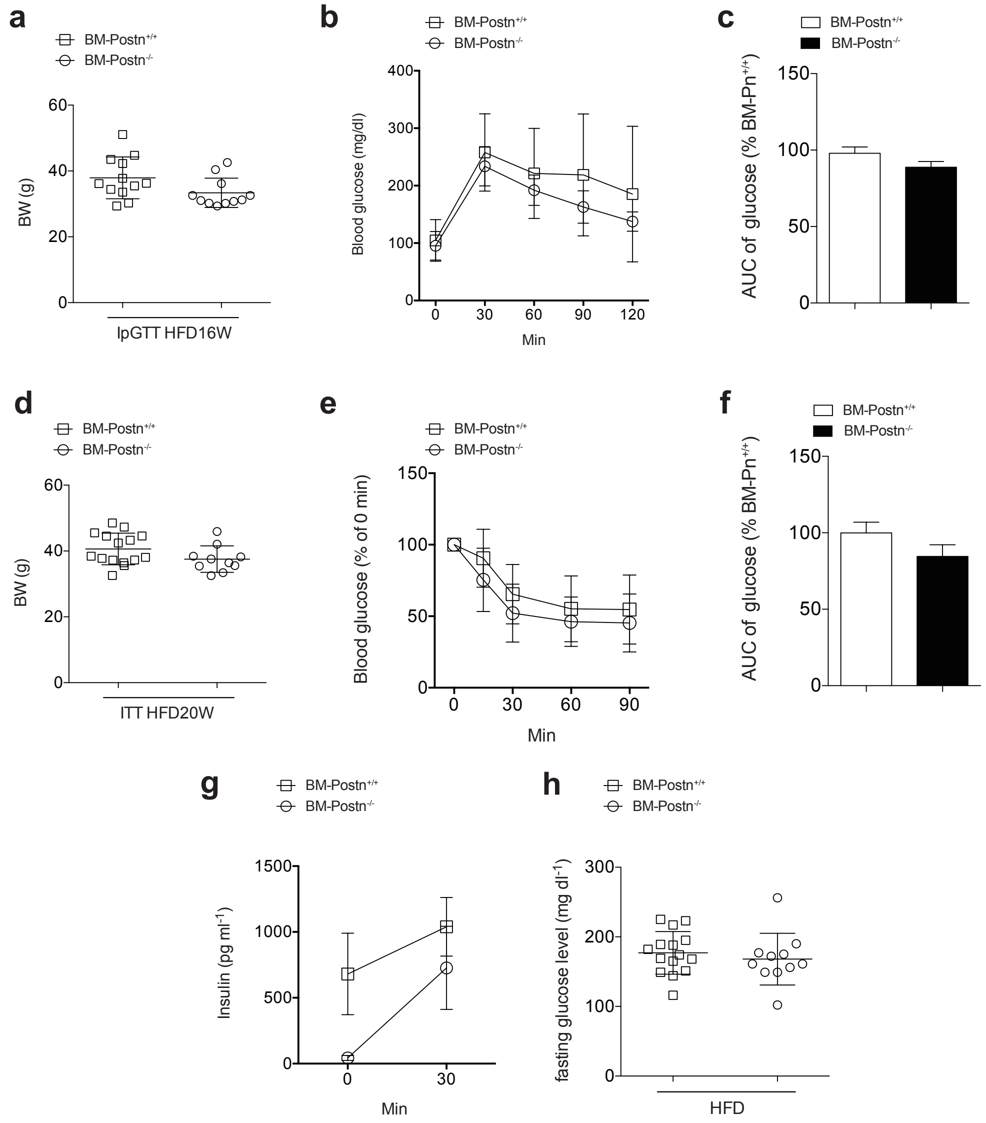

Supplementary Fig. 7. Analysis of glucose and insulin tolerance in BM transplanted mice.

(**a**) Body weight of BM transplanted mice used in IPGTTs. n=11 each, analyzed by unpaierd t-test. (**b**) Serial changes in glucose levels after intraperitoneal injection of glucose in BM transplanted HFD-fed mice. (**c**) Area under curve (AUC) of glucose levels after intraperitoneal injection of glucose in BM transplanted HFD-fed mice . n=11 each, analyzed by multiple t-test. (**d**) Body weight of BM transplanted mice in ITTs. n=11-15, analyzed by unpaierd t-test. (**e**) Serial changes in glucose levels after intraperitoneal injection of insulin in BM transplanted HFD-fed mice. n=11-15, analyzed by unpaierd t-test. (**f**) AUC of glucose levels after intraperitoneal injection of insulin in BM transplanted HFD-fed mice. n=11-15, analyzed by unpaierd t-test. (**g**) Serial changes in insulin levels after intraperitoneal injection of glucose in BM transplanted HFD-fed mice. n=5 each, analyzed by multiple t-test. (**h**) Fasting blood concentration of glucose in BM-*Postn*$^{+/+}$ and BM-*Postn*$^{-/-}$ HFD-fed mice. n=11-15, analyzed by unpaierd t-test.

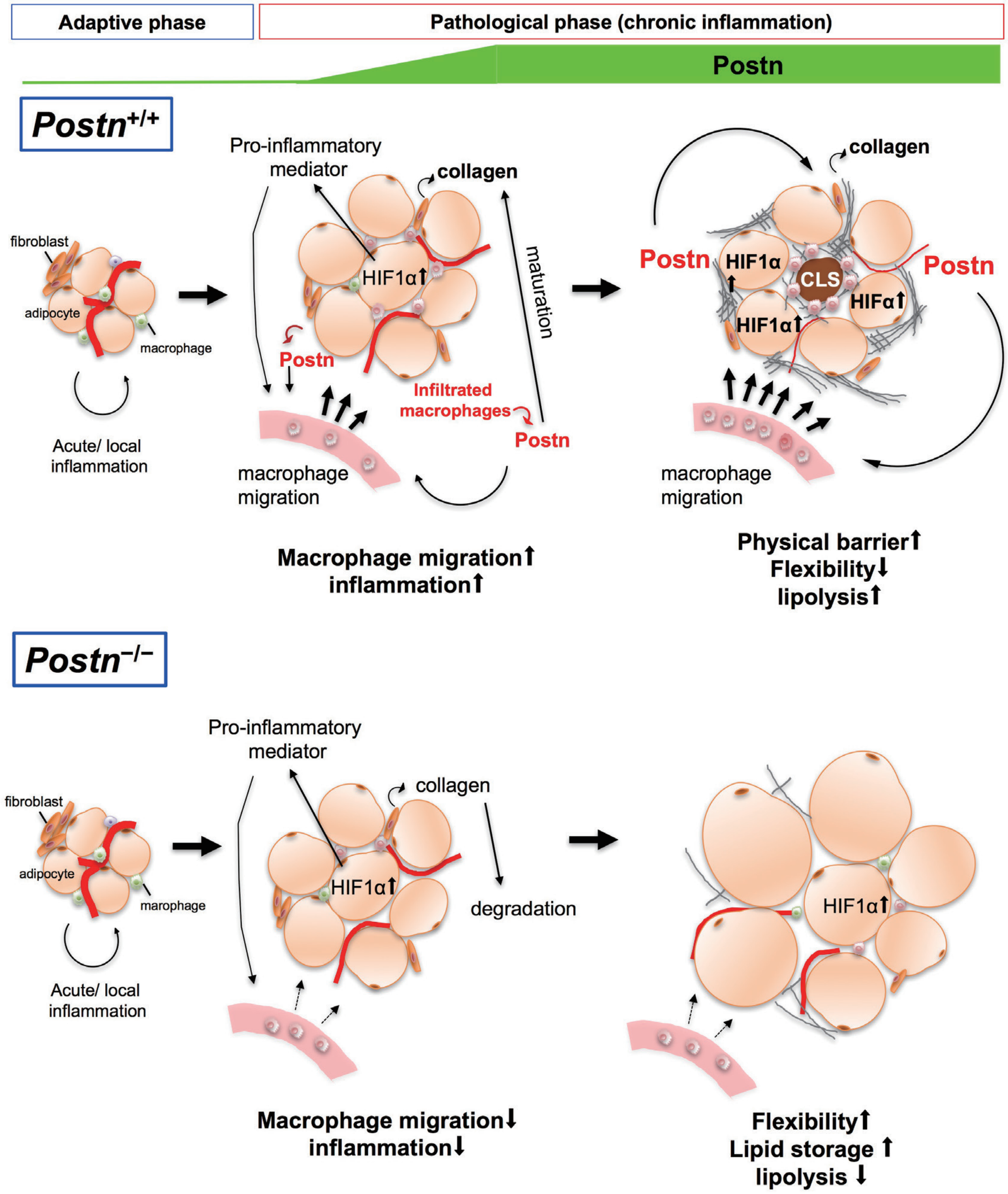

Supplementary Fig. 8. Potential role of Postn in obesity-induced adipose tissue inflammation and fibrosis.

During the development of obesity, Postn is produced in extraordinary amounts by adipose tissue. Postn was secreted at least from macrophages in visceral adipose tissue, possibly in response to hypoxia, and then sustained or amplified inflammation responses. Postn is required for the regulation of ECM production and maturation. Overproduction of ECM may limit the HFD-induced hypertrophy of adipocytes, which has a role in ectopic accumulation in the liver and systemic insulin resistance.
